# Optimization of Spark Plasma Sintering Technology by Taguchi Method in the Production of a Wide Range of Materials: Review

**DOI:** 10.3390/ma16165539

**Published:** 2023-08-09

**Authors:** Robert Kruzel, Tomasz Dembiczak, Joanna Wachowicz

**Affiliations:** 1 Faculty of Civil Engineering, Czestochowa University of Technology, 3 Akademicka Street, 42-200 Czestochowa, Poland; kruzel@bud.pcz.pl; 2 Faculty of Science and Technology, Jan Dlugosz University in Czestochowa, Armii Krajowej Street 13/15, 42-200 Czestochowa, Poland; 3 Department of Mechanical Processing of Wood, Institute of Wood Sciences and Furniture, Warsaw University of Life Sciences, Nowoursynowska Street, 166, 02-787 Warsaw, Poland

**Keywords:** powder metallurgy, spark plasma sintering, optimization Taguchi method

## Abstract

This paper reviews the production of sinters using the spark plasma sintering method. SPS (spark plasma sintering) technology has been used for several decades, mainly in laboratories, to consolidate a huge number of both new and traditional materials. However, it is now more often introduced into practical industrial use, with equipment as early as the fifth generation capable of producing larger-size components at competitive costs. Although the mechanism of sintering with the use of this method is not yet understood, the effectiveness of the SPS process for the rapid and efficient consolidation of a wide range of materials with novel micro-structures remains indisputable. With a relatively wide variation in chemical composition, the structure allows the selection of appropriate consolidation parameters for these materials. The influence on the values of apparent density and mechanical properties depends on the parameters of the spark plasma sintering process. In order to achieve a density close to the theoretical density of sinters, optimization of the sintering parameters, i.e., sintering temperature, heating rate, sintering time, pressing pressure and protective atmosphere, should be carried out. In this paper, the optimization of spark plasma sintering of Si_3_N_4_–Al_2_O_3_–ZrO_2_ composite was carried out using the Taguchi method. The effects of four sintering factors, namely heating rate, sintering time, sintering temperature and sintering pressure, on the final density were investigated. Optimal sintering conditions were proposed and a confirmation experiment was conducted. The optimal combination of sintering conditions for spark plasma sintering (SPS) of Si_3_N_4_–Al_2_O_3_–ZrO_2_ composite for high apparent density was determined as A3-B3-C3-D2. Based on ANOVA analysis, it was found that the apparent density of sintering was significantly influenced by sintering temperature, followed by pressing pressure, sintering time and heating rate. Validation of the developed mathematical model predicting the apparent density of sinters showed close agreement between the predicted response results and experimental results.

## 1. Overview of the SPS Process

The SPS method is used for sintering powder materials. It belongs to a broader group of current-activated methods—PECS (Pulsed Electric Current Sintering) [1,2,3,4,5,6,7]. This method is used for sintering various advanced composite materials (particle-reinforced, fiber-reinforced), ceramic materials (cermets, oxide and non-oxide ceramics, superhard materials), biomaterials and FGMs [8,9,10,11,12,13,14].

The first laboratory attempts to make a sintering device using pulses of electrical energy were carried out in the USA as early as 1933 [15,16,17,18]. In 1950, work on a method called Spark Sintering was started by Lenel [18,19]. Subsequently, leading achievements regarding the development of this technology were obtained by scientists from Lockheed Missile and Space Company in California [18,20] and Inoe from Japan [18,21,22]. Due to the high cost of the device and its uncommon use at the time, the method did not find wider application. A second-generation apparatus was developed in the late 1980s and early 1990s. At the time, the method was called Plasma Activated Sintering (PAS) and allowed for a maximum pressing force of 50 kN and a DC generator of up to 800 A [18]. The third generation of SPS equipment uses power supplies of up to 60,000 A and clamping forces of up to 3000 kN. The fourth generation of equipment are production systems adapted to medium-scale production [23]. The sample device parameters in the SPS system are summarized in Table 1.

### 1.1. Fundamental Principles of Spark Plasma Sintering

**Figure 1:**
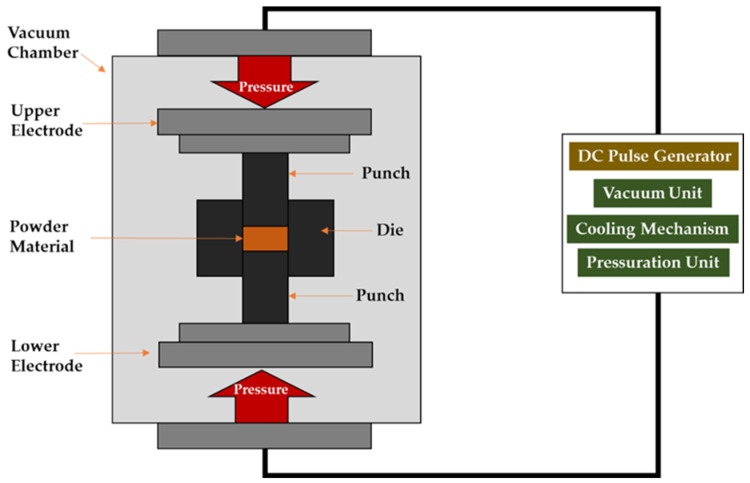
Diagram of the Spark Plasma Sintering Process.

In the SPS method, powder is placed in a graphite set (die + two punches) which constitutes a kind of heating element when an electric current flows through it. During the sintering process, the entire set is subjected to uniaxial pressure by means of water-cooled stamp electrodes. The sintering set is placed in a water-cooled vacuum chamber. The powder is heated by the Joule heat given off when current pulses flow through the die and sintered powder. Temperature measurement is carried out using pyrometers (axial/radial) or flexible thermocouples [26]. A schematic of a SPS-type device is shown in Figure 1.

### 1.2. Mechanisms Involved in SPS Sintering

The SPS process is carried out using electrical discharges of high intensity but low voltage (of a few volts). In theory, each newly generated pulse should flow along a different path between the powder particles. A plastic flowing effect of the material occurs due to the pressure applied between the punches. Combining this phenomenon with diffusion processes makes it possible to obtain a material with a porosity of less than 1% [18]. The exact mechanism of sintering, as well as the role of pulsed currents in electric field-assisted methods, are currently not fully clarified. Whether discharges and plasma are involved in the sintering process is still controversial. Hitchcock et al. [27] measured the voltage–time–frequency during sintering using an oscilloscope and demonstrated the absence of spark discharge and plasma during the whole process of SPS, whereas Zhang et al. [28] observed direct discharge phenomena and the effect of the discharge caused local high temperatures and plasma. Due to the difficulty of direct observation or provision of local high temperatures induced by discharges, finite element modeling (FEM) of the sintering process was found to be an effective method by adjusting the internal physical parameters to study the sintering mechanism.

In [29], the authors described the sintering mechanism by dividing it into the following stages:

(1) Activation and surface cleaning of powder particles;
(2) Formation of “necks” between the particles;
(3) Growth of the resulting “necks”;
(4) Compaction of the material as a result of its plastic deformation.

The work [30] investigated the mechanism of SPS sintering on a macro scale for ultrafine-grained copper. Studies have shown that, for the finest metal powders, the evolution of the micro-structure is highly dependent on the sintering parameters. There are always the four consolidation stages mentioned above. High-quality sinters can only be obtained if all sintering stages are followed in sequence. For fine copper powder, the optimized sintering parameters are selected as follows: sintering temperature, 750 °C; initial pressure, 1 MPa; pressure, 50 MPa; heating rate, 80 °C/min; and holding time, 6 min.

In their work [31], X. Song et al. studied the mechanism of neck formation and growth. They used spheroidal copper for the study. They carried out the sintering process at different temperatures (from 440–880 °C) and at a constant pressure—20 MPa. They deliberately used low pressures to eliminate the impact of plastic deformation on the growth and formation of necks. They achieved full sinter thickening by sintering at 880 °C with a heating rate of 110 °C/min. As the sintering temperature increased, the diameter and size of the necks increased, which was also reflected in the density. The particle contact diameters (necks), for processes carried out at temperatures 550 and 660 °C, were 1/7 and 1/5 of the average grain size, respectively. Additionally, observations were made of the neck break morphology. In the neck zone, they revealed fine grains which formed, most likely, as a result of local melting and subsequent rapid solidification of the particles’ surface. The temperatures of 550 and 660 °C are well below the melting point of Cu. However, the extremely high surface temperature, generated in the contact area of the particles, causes local melting of the material and the much lower interior temperature of the particles causes rapid solidification of the molten material due to rapid heat dissipation. A similar phenomenon, different from the conventional sintering mechanism, in the early stage of neck formation has also been observed for other materials. The main difference between conventional sintering and the SPS method is the heating rate of the material. The SPS method gives a heating rate of approximately 1000 °C/min.

In the SPS process, owing to the pulses of direct current flowing through the powder particles, their surface is more cleaned and activated, compared to conventional sintering methods. Therefore, high-quality sintering is obtained at a lower temperature and in a shorter time.

In the paper [32], pure magnesium was prepared by SPS and the mechanism of sintering densification was discussed by numerical simulation and simultaneous experimentation. The results show that a dense magnesium oxide layer was formed on the surface of the Mg particles, and the oxide reduction can be observed due to the removal effect of the oxide layer in SPS. The high-energy pulsed current flows in a preferential way across the particle interfaces, creating conditions for the formation of a micro-arc between the particles, and the temperature at the particle interfaces can reach up to 1979 °C. With low pressure at the initial sintering stage, the local high temperature induced by the micro-arc causes melting (and even evaporation) to occur.

### 1.3. The Basic Differences between Conventional Sintering and SPS

**Figure 2:**
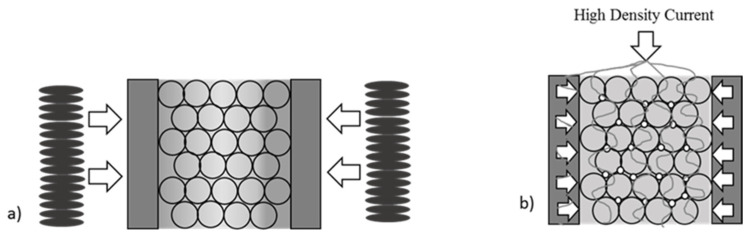
Heating mechanism of the material: (**a**) free sintering, (**b**) spark plasma sintering.

In the conventional sintering method, the material is heated from the sinter surface to the core (by radiation and convection). The heating rate and efficiency are, therefore, low. As a result, there is a significant temperature gradient inside the mold, which translates into inhomogeneities in the sintered material (Figure 2). The main disadvantage of this type of process is the need for higher temperatures and longer times to obtain a high-density sinter. These conditions affect grain growth in the consolidated sinter, affecting parameters such as tensile strength and hardness, among others. The high temperature of the sintering process and the long time required also cause problems when sintering materials with a high affinity for oxygen. The long time required also increases the cost of sinter production and limits the ability to carry out multiple sintering processes per day [33].

For example, in order to limit grain growth during the consolidation of WC–Co cemented carbides, alloying additives are used. In the work [34], grain densification and growth during heating to the sintering temperature were investigated. It was shown that the relative density of a nanocrystalline powder is 90% below the solidus temperature. This is likely due to the anisotropy of the surface energy of tungsten carbide, which can be modified by introducing alloying elements. Note that decreasing the nanoparticle size speeds up the compaction of the sinter.

The authors in the paper [35] observed that the original (10 nm) grain size of nanophase WC–Co alloy increased to 900 nm after sintering in a vacuum furnace at 1400 °C. Grains were observed to start rapidly growing almost immediately after the beginning of heating (no holding). There is a critical temperature above which grains start growing dramatically.

It is well known that the hardness of hard metals is inversely proportional to the grain size. However, the relationship between hardness and fracture toughness may be non-linear depending on grain size. The smaller the grain size, the lower the fracture toughness. However, nanostructured alloys have other hardening mechanisms: the grain boundaries inhibit cracking, which enhances fracture toughness. In the case of WC-Co carbides, the fracture toughness is improved when the grain size of the WC phase reaches a value of several nanometers [36].

Sinters containing nano-sized grains also have better mechanical properties. They have smaller defects (including, of course, pores), which is beneficial for high fracture toughness. It should be noted that the properties of nanostructured hard metals such as hardness, flexural strength, wear resistance and fracture resistance are improved. However, the fracture toughness and the number of thermal cracks deteriorated. However, grain size has no effect on density, modulus of elasticity, thermal shock resistance or thermal expansion [37].

Unconventional sintering techniques were developed primarily to limit unfavorable grain growth. Most authors point out that nanoparticles can be obtained after sintering only by applying very high pressure [38].

### 1.4. Influence of Temperature Distribution during SPS

In SPS-type methods, the powder is heated relatively uniformly throughout the volume. The increase in temperature occurs through the generation of Joule heat, as a result of the flow of electric current pulses through the sintered powder (direct heating). As temperature plays a fundamental role in SPS sintering processes, it is necessary to precisely determine the temperature distribution on the individual components of the sintering set-up. Having detailed information about the temperature distribution makes it possible to control the SPS parameters, optimize the sintering process and ultimately improve the quality of the materials produced [1,8]. Several experimental studies have been carried out to obtain efficient and optimized temperatures during the sintering process. However, the experimental procedures provide temperature readings at specific points only. Numerical methods can provide detailed information about the temperature at any point [39,40].

In [41], the temperature distribution during sintering of Al_2_O and ZrB_2_ samples was obtained using the finite element method. The results showed that, in the case of non-conductive Al_2_O materials, the current concentration on the graphite matrix near the sample is high, resulting in a higher matrix temperature compared to the conductive matrix. In contrast, a more uniform current distribution is observed during the sintering of ZrB_2_. The maximum temperature is observed on the punch. The electric current diagram strongly depends on the electrical properties of the sintered materials. The temperature distribution in the graphite matrix is almost linear in both non-conductive and conductive materials. The temperature distribution is more uniform for non-conductive materials, compared to conductive materials, resulting in more homogeneous properties of the baked sample.

Experimental and numerical studies were carried out in the work [42] to investigate temperature heterogeneity depending on the SPS sintering equipment used during silicon nitride consolidation. A new punch geometry was proposed, while leaving the remaining SPS parameters constant. The study showed that the geometry changes provided an efficient methodology to direct the flow of electric current, able to redistribute temperatures in the powder.

### 1.5. The Effect of the Sintering Atmosphere

The composition of the sintering atmosphere and the partial pressure of its constituents have an extremely important influence on the properties of the obtained sinter (phase stability, degree of oxidation, grain growth). In [43], silicon nitride was sintered in a hyperbaric nitrogen atmosphere. It was observed that the kinetics of the intermediate densification step is very sensitive to the sintering atmosphere and densification time. In a previous study, lead-free piezoelectric ceramics were sintered in o2 and H2 atmospheres and the influence of the atmosphere on grain growth was investigated. Sintering in a reducing atmosphere delayed the onset and reduced the amount of abnormal grain growth.

Sintering of metallic systems requires a protective atmosphere to prevent oxidation. In addition to avoiding undesired reactions, the atmosphere should also remove reaction products, e.g., from the evaporation of lubricants (e.g., paraffin), or the reduction of oxides from the grain surface of powders. As far as oxygen is concerned, the atmosphere should be inert or reducing, and there is also the possibility of carburizing and decarburizing the atmosphere. In some cases (e.g., stainless steel), it is worth considering the effect of nitriding. For WC–Co composite materials, control of the carbon content is the key to obtaining good materials.

As far as the sintering of hard metals and highly reactive materials is concerned, the sintering process is carried out under vacuum. A low partial pressure of oxygen must be ensured during the process. By reducing the gas pressure in the sintering chamber, surface contamination of the powder particles is reduced. In addition, a vacuum is used to avoid reactions with nitrogen, hydrogen or oxygen, especially in the case of metallic materials.

In paper [44], the authors report the densification of Ca_0.9_Yb_0.1_MnO_3_−x ceramics by SPS in different atmospheres and using different tools. Studies have shown that airborne processing is possible. The thermoelectric properties of the SPS-treated sample in air are similar to those of materials obtained by conventional sintering.

In another paper [45], the effect of the sintering atmosphere on the micro-structure and mechanical properties of Ti_2_AlN was investigated. The powder mixtures were subjected to mechanical activation in an argon atmosphere, followed by SPS sintering in a vacuum or nitrogen atmosphere. The use of nitrogen as a reactive sintering atmosphere did not favor the spark plasma sintering condition of the powder mixture of Ti and AlN. An excessive formation of the secondary TiN and Ti_4_AlN_3_ phases resulted. Qualitative analysis of the diffraction peaks of the Ti_2_AlN MAX samples showed that the use of a nitrogen atmosphere caused a significant change in the phase composition of the samples.

SiC–ZrB_2_ composites were also produced using the SPS method in an argon atmosphere. No reaction was observed between β-SiC and ZrB_2_. For the best variant, a density of 97% and mechanical properties of 100 MPa were obtained [46].

## 2. Optimization of Sintering Parameters during the Manufacture of Composites Using Spark Plasma Sintering (SPS) Technology

### 2.1. Introduction to Optimization

During industrial transformation, the implementation of new manufacturing technologies is an integral part of the effort to usher in the next great industrial change. High manufacturing efficiency at low cost, with maintenance, and with high quality are the main goals of most manufacturing companies. Validation of optimized production processes in an industrial setting enables the improvement of a specific operation while minimizing waste and reducing working time. In the most general terms, optimization consists in carrying out activities or processes in such a way that the end result can be obtained with the least expenditures while maintaining quality characteristics.

The scientific literature is devoted to many studies on the planning and optimization of experiments. Design of experiments (DOE) allows you to identify process conditions that affect quality and determine the values of input variables (factors) to guarantee the best results. Experiments consist of runs (tests) in which changes are made to input variables or factors. Of the many experimental methods, the Taguchi method deserves special attention. The goal of the Taguchi method is to optimize the quality of multi-parametric technological processes. In the Taguchi method, in the first stage, the process parameters should be determined; these parameters will then be the input quantities of the experimental plan. In the next stage, you need to select an orthogonal array suitable for the experiment. In the next stage, decide how the characteristics that determine quality are measured. In the final stage, research should be carried out and the quantities that most strongly affect the S/N ratio should be identified.

The application of the design of experiments (DOE) method in powder metallurgy technology, which is essentially an experimental science, seems justified by increasing the efficiency of the process during the manufacture of new functional composite materials.

### 2.2. Product Optimization with the Use of the Taguchi Method

Optimization of product manufacturing using the Taguchi method is widely used in various industries, providing improvement of the quality of products on production lines. Gen’ichi Taguchi a Japanese engineer and statistician who used the concept of statistics and improved issues of quality from an engineering perspective. He stated that the overriding capability of a process should include not only the production of efficiency but should also comply with the target value. Any deviation from the target results in a loss of quality and a decrease in customer satisfaction [47].

Based on quality characteristics and variability, the Taguchi method uses the S/N ratio of the mean value (signal) to the standard deviation (noise). Quality features are divided into three types, nominal—the best; smaller—the better; and larger—the better. The S/N ratio is calculated according to the corresponding formula. The higher the S/N ratio, the lower the noise [48]. Antony et al. point to the low variability of the process and excellent quality stability [49]. In the Taguchi method, excellent quality is represented by the robustness of the process, indicating quality characteristics. Therefore, the Taguchi method is called “robust quality design” by some researchers and uses orthogonal arrays to configure experiments [50].

Wang et al., for example, used the Taguchi method to improve the thermomechanical reliability of flip chip packages at the wafer level [51]. Su et al. used the Taguchi method to optimize wire bonding quality parameters. The optimization allowed savings that amounted to about USD 0.7 million, which translated into a 0.8% increase in production efficiency [52]. Su et al. used Taguchi’s dynamic method to calculate the optimal setting of control coefficients and made sure that the result was close to the target value of optical white [53]. Limon-Romero, et al. used the Taguchi method to determine the optimal parameters for the tube cutting process in medical manufacturing [7]. Azadeh, et al. determined optimal parameters for space heating and cooling applications [54].

Among the above applications of the Taguchi method, there are also many scientific studies devoted to optimizing the parameters of the spark plasma sintering manufacturing process. Authors Sivakandhan et al. carried out optimization of the SPS sintering process to obtain Al 7068 alloy reinforced with tungsten carbide (WC) used in aviation. The input data were sintering temperature, pressing pressure and sintering time. Tensile strength results were the answer. Based on the analysis of variations, they found that sintering temperature had a dominant effect on tensile strength, while the other process parameters, i.e., pressing pressure and sintering time, had a less significant effect on tensile strength. The optimal sintering process parameters were sintering temperature of 550 °C, pressing pressure of 30 MPa and sintering time of 20 min [55].

Meanwhile, Balaka et al. used the Taguchi L_32_ experimental design to optimize the flexural strength of ZrB_2_-based composites produced by SPS sintering. For this purpose, they tested ZrB_2_-based composites to obtain a target experimental design with nine factors (SiC, carbon fiber (Cf), MoSi_2_, HfB_2_ and ZrC content, Cf(M.t, h) milling time and SPS parameters such as sintering temperature, sintering time and pressing pressure) at four different levels. The optimal experimental conditions were determined by statistical analysis of variance (ANOVA), and it was found that the most significant influence on the compaction of the sintered composite is temperature at 23.8%, while the other factors are also very important and influence results to a lesser extent: ZrC, 16.2%; HfB2, 9.8%; SiC, 9.6%; Cf, 8%; and MoSi_2_, 2%. Other factors, such as sintering time, Mt and pressing pressure, contribute to 6.2%, 5.7% and 3.9% of flexural strength, respectively [56].

Althahban et al. performed optimization of sintering temperature, pressing pressure, sintering time and heating rate to produce a tungsten carbide/silicon nitride/AA2219 composite. They designed experiments and performed analysis of variance to evaluate the effects of factors on density and microhardness. The optimal SPS sintering parameters were a sintering temperature of 500 °C, a pressing pressure of 30 MPa, a sintering time of 12 min and a heating rate of 160 °C/min, which translated into a density of 2.71 g/cm^3^ and a microhardness of 38.61 HV [57,58,59,60,61].

## 3. Optimization of Sintering Parameters in SPS Technology by Taguchi Method Based on Own Research

The purpose of this study was to optimize the consolidation of Si_3_N_4_–Al_2_O_3_–ZrO_2_ composite by SPS sintering using design of experiment (DOE) and analysis of variance (ANOVA). The effects of four sintering factors, namely heating rate, sintering time, sintering temperature and sintering pressure, on the final density were investigated. Optimal sintering conditions were proposed and a confirmation experiment was conducted.

### 3.1. Materials and Method

#### 3.1.1. Design of the Experiment

The sintering process was carried out in consideration of the following conditions: sintering temperature (A, 1550–1750 °C), heating rate (B, 100–200 °C/min.), sintering time (C, 300–900 sek.) and pressure (D, 40–50 MPa). For the four sintering factors, each factor was designed with three levels, as shown in Table 2. This is because the influence of these factors on the result may vary nonlinearly. An L_9_ (3^4^) orthogonal array (OA) was employed as it was able to provide the minimum degrees of freedom required for the experimental exploration. The column assignment and the experimental layout are shown in Table 3. After the completion of all experiments and the analysis of the data, a confirmatory experiment was performed at the recommended settings. This is an important step in Taguchi’s parameter design as it provides indications on the validity of the experimental procedures and results.

#### 3.1.2. Methodology of Material Preparation and SPS Sintering Process

The materials used for the study were ceramic powders with chemical compositions of Si_3_N_4_—5 wt%, Al_2_O_3_—5% by weight and ZrO_2_. The prepared powder mixture was mixed in a Turbula mixer for 24 h using beads at a ratio of 1:2.

**Figure 3:**
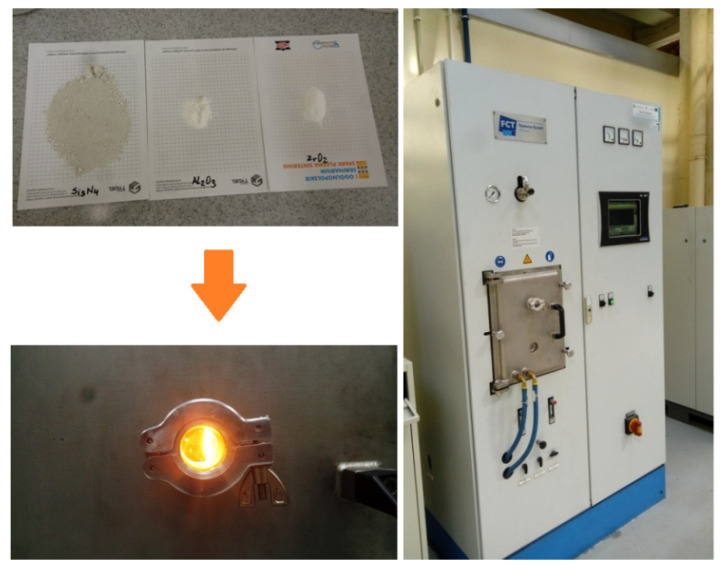
View of the spark plasma sintering furnace (FCT Systeme GmbH, Frankenblick, Germany) used to produce the ceramic composite [Proprietary development].

The sintering process was carried out using an FCT Systeme spark plasma sintering furnace (Figure 3). Sintering in vacuum was carried out using tools made of graphite. The feed chamber in the graphite tool assembly was filled with powder. A graphite film was placed between the powder, the die and the punch. As a result of the sintering process, sinters with dimensions of ϕ 20 × 3 mm were obtained.

#### 3.1.3. Density Measurement

**Figure 4:**
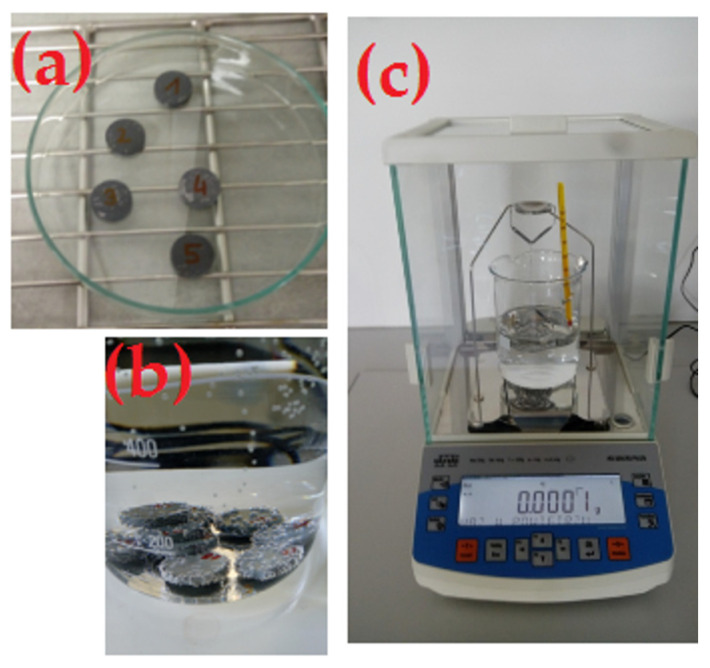
Density measurement by the Archimedes’ method according to EN ISO 623-2: 2001: (**a**) drying of samples in a dryer, (**b**) saturation of samples in liquid, (**c**) measurement on a balance [Proprietary development].

The apparent density was measured by hydrostatic weighing using Archimedes’ law in accordance with PN-EN ISO 623-2: 2001. A RADWAG AS 220.R2 PLUS laboratory balance (Radwag, Radom, Poland) was used for the measurements. The methodology for measuring the apparent density of the manufactured ceramic composites using the Arichemedes method is shown in Figure 4.

#### 3.1.4. Signal-to-Noise Ratio (S/N)

In sintering processes, a very important parameter of quality characteristics is the density of the sinter on which the mechanical properties of the sinter later depend. If the density of the sinter is close to the theoretical density (solid material) then the material is characterized by very good mechanical and functional properties. In the present study, the quality characteristics of sinters produced using the spark plasma sintering method is the apparent density. Hence, Equation (1) was used to calculate the S/N ratio and the results are shown in Table 5. Taguchi analysis was performed using Minitab 19.0 software and the mean S/N ratio plots were obtained; results, including those of analysis of variance (ANOVA), are presented in the following section.

Signal-to-noise ratio; the larger, the better:

(1) $$S/N={-10log}_{10}\left(\frac{1}{n}\sum_{i=1}^{n}\frac{1}{y_{i}^{2}}\right)$$

where n—no. of observations, y—observed data for each response.

### 3.2. Results and Discussion

Table 4 shows the results of measurements of the apparent density of the obtained sinters according to the experimental plan L_9_ (3^4^).

**Figure 5:**
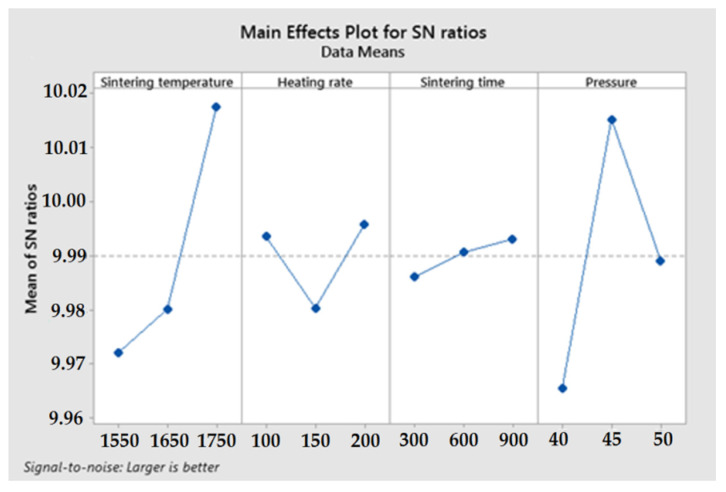
Mean Signal-to-Noise Ratios based on sintering test results.

Table 5 shows the S/N ratio responses for sinter densities obtained by the SPS method. It is noted that the highest average S/N ratio, which represents the minimum deviation difference between the desired output and the measured output, was obtained for a sintering temperature of 1750 °C, a heating rate of 200 °C/min, a sintering time of 900 sec. and a pressing pressure of 45 MPa. The predicted optimal combination of sintering parameters according to Figure 5 is shown as A3-B3-C3-D2 for Si_3_N_4_–Al_2_O_3_–ZrO_2_ composite produced by spark plasma sintering (SPS).

Analysis of variance (ANOVA) was conducted to determine the significant parameters of the spark plasma sintering (SPS) process. Table 6 shows the results of the ANOVA. The analysis of variance shows that the parameter sintering temperature had the greatest influence on the final density of the Si_3_N_4_–Al_2_O_3_–ZrO_2_ composite. The other parameters had less influence on the final density of sinters obtained by spark plasma sintering.

**Figure 6:**
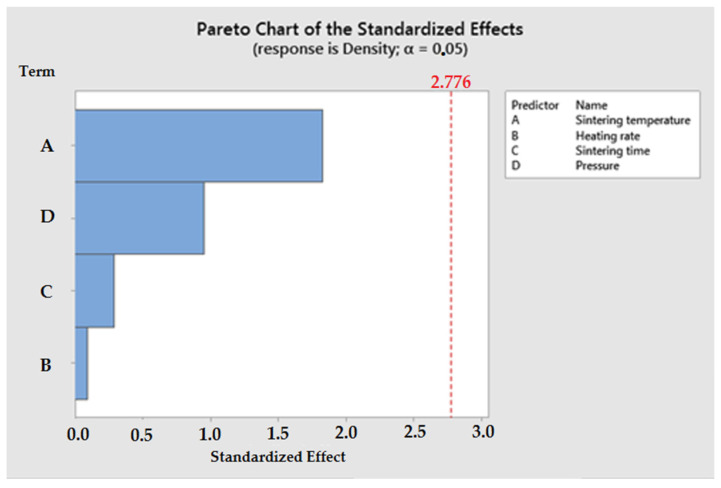
Pareto chart showing the influence of the most important factors on the consolidation process of ceramic composites using the Spark Plasma Sintering method.

The statistical significance of parameters affecting the spark plasma sintering (SPS) process of the Si_3_N_4_–Al_2_O_3_–ZrO_2_ composite is shown in the Pareto chart (Figure 6).

Based on linear regression, a mathematical model was developed to predict the apparent density of sinters produced by spark plasma sintering. The mathematical model was developed as a function of sintering temperature, heating rate, sintering time and pressing pressure. The equation obtained from the linear regression analysis is presented as follows:

**Density** = 2.9789 + (0.000083 × A) + (0.000008 × B) + (0.000004 × C) + (0.000863 × D) (2)

where A—sintering temperature, B—heating rate, C—sintering time, D—pressure.

Analysis of the quality of the developed mathematical model was carried out using the coefficient of determination R^2^. The coefficient of determination indicates how much of the variability (variance) in the sample coincides with the correlations included in the model. The coefficient of determination (R^2^) is, therefore, a measure of the degree to which the model fits the sample. The coefficient of determination takes values in the range (0, 1). The fit of the model is better the closer the R^2^ value is to unity.

**Figure 7:**
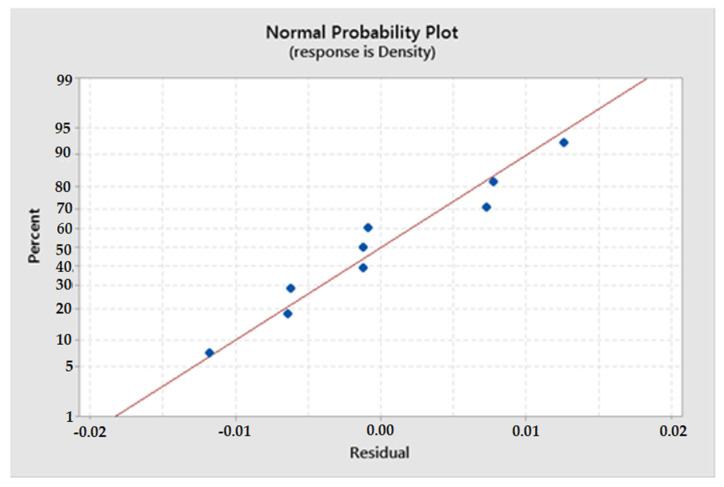
Normal probability plot of the residuals for apparent density.

In this study, the developed regression model has a coefficient of determination of R^2^ = 51.97%. Based on the residual plot, the significance of the determined coefficients in the predicted model was determined. In Figure 7, it is observed that the residual graph is a straight line, which means that the residual errors in the model have a normal distribution, and the determined coefficients in the model are significant.

**Figure 8:**
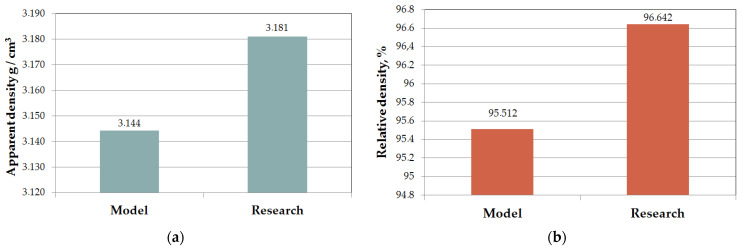
Validation of the developed mathematical model describing the density of Si_3_N_4_–Al_2_O_3_–ZrO_2_ composite: (**a**) apparent density, (**b**) relative density.

A confirmation test was conducted to validate the developed mathematical model’s prediction of apparent densities. The process of spark plasma sintering (SPS) to produce Si_3_N_4_–Al_2_O_3_–ZrO_2_ composite was carried out according to the following parameters: sintering temperature 1400 °C, heating rate 300 °C/min, sintering time 900 s and a pressing pressure of 50 MPa. The powder mixture preparation process and density measurements were carried out according to the methodology described earlier in this paper. Figure 8 shows the model’s results and experimental verification.

Based on the results of the validation of the mathematical model, it was found that the predicted results from the model and the experimental results agreed within the given range of parameters.

It was also found that the mathematical model predicted the apparent density with a small error of only 1.555% compared to experimental tests at a sintering temperature of 1400 °C and a heating rate of up to 300 °C/min. Such an arrangement of parameters has not been analyzed before; thanks to such an arrangement, 96.642% of the theoretical sinter density and a hardness of 1778.2 HV10 was achieved while reducing the energy consumption required for the SPS sintering process.

Using the Minitab 19.0 software tool for statistical modeling, contour plot analysis was conducted. The purpose of the analysis was to examine the relationship between the response variable and the two control variables by depicting the contours of the predicted response variables.

**Figure 9:**
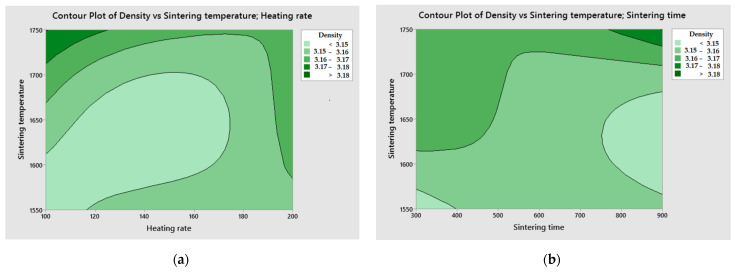

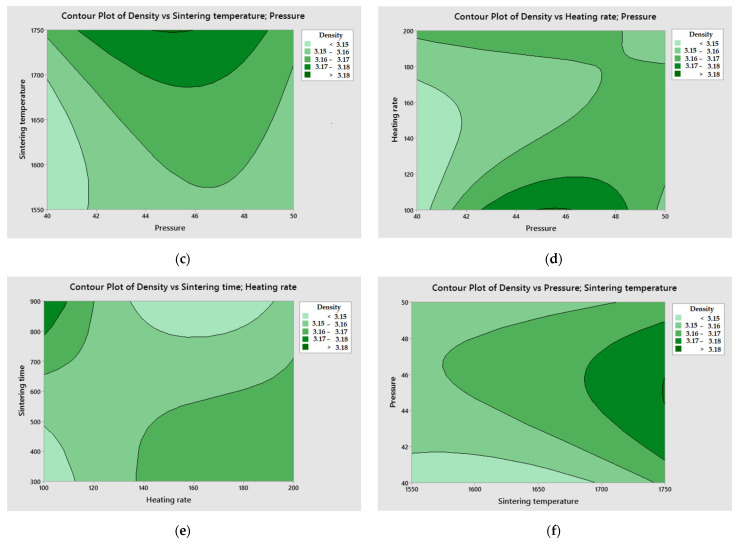
Contour plot of apparent density of Si_3_N_4_–Al_2_O_3_–ZrO_2_ composite sinter: (**a**) sintering temperature vs. heating rate, (**b**) sintering temperature vs. sintering time, (**c**) sintering temperature vs. pressing pressure, (**d**) heating rate vs. pressing pressure, (**e**) sintering time vs. heating rate, (**f**) pressing pressure vs. sintering time.

Figure 9 shows the contour plots explaining the relationship between the sintering process parameters and the apparent density of the sinter.

Figure 9 shows contour plots explaining the relationship between the parameters of the sintering process and the apparent density of the sinter.

From Figure 9a, it was found that high apparent density of sinters can be obtained in two stages. The first is by increasing the sintering temperature above 1700 °C and with an extended heating time of 100 °C/min. The second lies in lowering the sintering temperature below 1600 °C while reducing the sinter heating time to 200 °C/min. From Figure 9b, it was found that increasing the sintering temperature and increasing the sintering time generate an increase in the apparent density of the sinter. In Figure 9c, it is observed that high values of sinter density can be obtained below a sintering temperature of 1700 °C, assuming that the value of pressing pressure increases. In Figure 9d, it was observed that good sinter density values can be obtained by increasing the sinter heating time while increasing the sinter pressing pressure. In Figure 9e, it is observed that it is possible to reduce the sintering time and shorten the sinter heating time to generate sinter density at a good level. In Figure 9f, it is observed that as the sinter pressing pressure increases and the sintering time increases, the apparent density of the sinter also increases.

## 4. Conclusions

Given the increasingly significant role of energy in the cost of manufacturing, reducing energy consumption is becoming a primary goal for scientific and industrial communities. It is estimated that reducing energy consumption by one megawatt per week could save approximately USD 1 million per year. In doing so, it is necessary to take into account the negative effects that the electricity production and distribution process has on the surrounding environment. Problems of excessive energy demand have led to the optimization of production processes in minimizing energy consumption. The way to meet this challenge at the manufacturing process level is to optimize productivity while maintaining high product quality and relatively low energy consumption.

Powder metallurgy is one of the main methods for obtaining modern ceramic materials, metallic materials and metal–ceramic composites. Scientific progress in the area of new-generation materials has become one of the most important issues in modern materials engineering.

The current state of the art in the production of new materials using spark plasma sintering and Taguchi method optimization of sintering parameters, i.e., sintering temperature, heating rate, sintering time, pressing pressure and protective atmosphere, makes it possible to achieve a final sinter with a high apparent density close to the theoretical density with relatively high mechanical properties.

Based on optimization of the Taguchi method spark plasma sintering process, the following conclusions were drawn:

- The optimal combination of spark plasma sintering (SPS) conditions of Si_3_N_4_–Al_2_O_3_–ZrO_2_ composite for obtaining high apparent density was determined as A3-B3-C3-D2;
- Based on ANOVA, it was observed that the apparent density of sinters was significantly affected by the sintering temperature, followed by the pressing pressure, sintering time and heating rate;
- From the point of view of a developed mathematical model for the apparent density, a close correspondence was observed between the predicted response results and the experimental results. Thus, the developed models can be used to properly select the process conditions for spark plasma sintering of Si_3_N_4_–Al_2_O_3_–ZrO_2_ composite without the need for experimental testing;
- Thanks to the developed mathematical models, it is possible to reduce the cost of sintering production by optimizing the parameters of the consolidation process.

## Figures and Tables

**Table 1:** Configuration of a typical SPS system [24,25].

Max. temperature attainable lies in the range of 1800–2200 °C
Hydraulic press capable of applying a force in the range of 10–400 tons
Heating rate of 5–1000 K/min (depending on tool size)
Holding time ~2–30 min
Pulsed DC power source providing a current in the range of 3000–40,000 A at 1–20 V
Max. power capacity rating of 37–1200 kW
Vacuum in cold oven 5 × 10^−2^ mbar
Process gases—Ar, N_2_ (max. 5 bar)
Pulse on/off is freely programmable (1...999 ms) for each individual segment

**Table 2:** Factors and levels for sintering experiments.

Symbol	Process Parameters	Units	Level 1	Level 2	Level 3
A	Sintering temperature	°C	1550	1650	1750
B	Heating rate	°C min^−1^	100	150	200
C	Sintering time	sek.	300	600	900
D	Pressure	MPa	40	45	50

**Table 3:** Experimental plan according to the orthogonal array L_9_ (3^4^).

No. Exp.	Sintering Temperature (A)	Heating Rate (B)	Sintering Time (C)	Pressure (D)
1	1	1	1	1
2	1	2	2	2
3	1	3	3	3
4	2	1	2	3
5	2	2	3	1
6	2	3	1	2
7	3	1	3	2
8	3	2	1	3
9	3	3	2	1

**Table 4:** Results of density measurements of the obtained sinters.

No. Exp.	Theoretical Density g/cm^3^	Apparent Density g/cm^3^	Relative Density %
1	3.292	3.143	95.476
2		3.158	95.932
3		3.155	95.842
4		3.156	95.878
5		3.144	95.498
6		3.165	96.143
7		3.180	96.612
8		3.163	96.097
9		3.162	96.056

**Table 5:** Response table for Signal-to-Noise Ratios.

Level	Sintering Temperature	Heating Rate	Sintering Time	Pressure
1	9.972	9.994	9.986	9.965
2	9.980	9.980	9.991	**10.015**
3	**10.018**	**9.996**	**9.993**	9.989
Delta	0.046	0.016	0.007	0.050
Rank	2	3	4	1

**Table 6:** Analysis of Variance.

Source	Degrees of Freedom	Sum of Square	Mean Square	F-Value	*p*-Value
Regression	4	0.000537	0.000134	1.08	0.471
Sintering temperature	1	0.000414	0.000414	3.34	0.142
Heating rate	1	0.000001	0.000001	0.01	0.933
Sintering time	1	0.000010	0.000010	0.08	0.789
Pressure	1	0.000112	0.000112	0.90	0.397
Error	4	0.000497	0.000124	-	-
Total	8	0.001034	-	-	-
